# The Anti-Vivisection Hospital

**Published:** 1909-08-28

**Authors:** 


### THE ANTI-VIVISECTION HOSPITAL.

THE correspondence, which we publish this week, between Sir Henry Burdett and the Secretary of the Anti-Vivisection Hospital is a good example of the difficulty of getting a plain answer to a plain question. It is true that the question was implied, not spoken; still, it was a plain question. The hospital was charged, at a public meeting, with Pretentious Humbug. What is Pretentious Humbug? To treat a case of rabies with a " Buisson bath," to treat a case of laryngeal diphtheria without anti-toxin, to call that anti-toxin " animal filth," to advertise " No experiments on hospital patients " when the whole hospital is an experiment, to advertise " No vivisection in its school " when there is no school—these acts are of the nature of Pretentious Humbug. The question is plain as plain can be.

That a Buisson bath " for the prevention or cure of hydrophobia " is, or was, installed at the Anti-Vivisection Hospital is certain; it is certain, also, that this absurd bath has been puffed, as it were Mother Seigel's Syrup, for its power of " drawing the poison out of the veins." That the senior physician of the hospital about a year and a-half ago called diphtheria anti-toxin " animal filth " at a huge public meeting in the Battersea Town Hall was reported in the *Times*. That the hospital does not admit diphtheria cases, and would not give them anti-toxin, may be gathered from the evidence given by one of its staff before the Royal Commission. That experiments are indeed made upon its patients may be gathered from a speech made by another member of its staff; he is reported to have said that one member of the staff was a homœopathic practitioner, and that they " worked well together," or words to that effect. How can a regular practitioner and a homœopathic practitioner work well together without experimenting on a patient?

Again, the general contempt which is felt in the medical profession for this hospital is strengthened by the outspoken statement made at the Mansion House meeting by Mr. Bryant, and by the senseless quarrelling which from time to time cannot be hid within the hospital, and finds its way into the public papers.

With regard to the special instances taken by Sir Henry Burdett, we have, for Lord Lister's work, Lord Lister's own statement, that his work, till he " welcomed Pasteur's demonstration " was " all in vain." And what was Pasteur's demonstration, but the demonstration, in animals, of the specific causes of wound-infection? For the testing of the sputa for tubercle-bacilli, which, happily, is done at the hospital, we can only express our amazement that the hospital should use this test, and should still proclaim itself as opposed to all experiments on animals. For it is absolutely certain that the specific cause of phthisis was proved by experiments on animals, and without them could not have been proved.

It is reported that they who are directly responsible for the continued existence of this hospital have refused the grant which was made to it by the Metropolitan Hospital Sunday Fund. The grant was accompanied with hard words such as leave a black mark that does not soon come out. All the same, there is no present evidence that the hospital will " mend its ways, purge its methods, and fall into line " with better hospitals. It has made its bed, and must lie on it. Slowly, but surely, the cause for which it is supported by kind-hearted people is losing strength among the educated classes. The hope is that the hospital, in years to come, will quietly drop its present name, and make other necessary changes in its general behaviour; and so, at last, win from the medical profession that confidence which is given to other hospitals.

